# A synthesis of the patho-physiology of *Mycobacterium avium* subspecies *paratuberculosis* infection in sheep to inform mathematical modelling of ovine paratuberculosis

**DOI:** 10.1186/s13567-018-0522-1

**Published:** 2018-03-07

**Authors:** Nelly Marquetoux, Rebecca Mitchell, Anne Ridler, Cord Heuer, Peter Wilson

**Affiliations:** 1 EpiCentre, Institute of Veterinary, Animal and Biomedical Sciences, Massey University, Palmerston North, New Zealand; 2 Department of Mathematics and Computer Sciences, Emory College of Arts and Science, Atlanta, GA USA; 3 Department of Population Medicine and Diagnostic Sciences, Cornell University, Ithaca, NY USA; 4 Institute of Veterinary, Animal and Biomedical Sciences, Massey University, Palmerston North, New Zealand

## Abstract

This literature review of exposure to *Mycobacterium avium* subsp. *paratuberculosis* (MAP) in sheep enabled a synthesis of the patho-physiology of ovine paratuberculosis (PTB). These results could be used to inform subsequent modelling of ovine PTB. We reviewed studies of both experimental and natural exposure. They were generally comparable. Possible outcomes following exposure were latent infection, i.e. mere colonization without lesions; active infection, with inflammatory histopathology in the intestinal tissues resulting in mild disease and low faecal shedding; and affection, with severe intestinal pathology, reduced production, clinical signs and high faecal shedding. Latent infection was an uninformative outcome for modelling. By contrast, histological lesions and their grade appeared to be a good marker of active infection and progression stages to clinical disease. The two possible pathways following infection are non-progression leading to recovery and progression to clinical disease, causing death. These pathways are mediated by different immune mechanisms. This synthesis suggested that host-related characteristics such as age at exposure and breed, combined with pathogen-related factors such as MAP dose, strain and inoculum type for experimental infection, have a strong influence on the outcome of exposure. The material reviewed consisted of disparate studies often with low numbers of sheep and study-level confounders. Hence comparisons between and across studies was difficult and this precluded quantitative model parameter estimation. Nevertheless, it allowed a robust synthesis of the current understanding of patho-physiology of ovine PTB, which can inform mathematical modelling of this disease.

## Definitions

In the literature, PTB is classically defined as a chronic granulomatous enteritis caused by infection of the intestinal tract with MAP. In this review, more specific terminology is used as follows to disambiguate the various terms related to the epidemiology and infection with MAP.

Paratuberculosis is used as a generic term referring to all aspects of epidemiology and infection with MAP, encompassing:

- Latent infection: the presence of MAP in host tissues without signs of disease (i.e. no pathology or symptoms of disease), synonymous with mere colonisation. Latent infection can thus only be detected by identifying the presence of MAP, i.e. by culture or PCR of ileal tissue and/or mesenteric lymph nodes;
- Active infection: the presence of pathology caused by MAP, whether subclinical or resulting in clinical disease. Sheep with active infection are referred to as Affected.
- Clinical disease: clinically affected, usually a fatal wasting condition also called Johne’s disease, concerning a subgroup of sheep experiencing active infection.

## Introduction

Ovine paratuberculosis (PTB) is the infection of the intestinal tract of sheep with *Mycobacterium avium* subsp. *paratuberculosis* (MAP) that can cause a fatal wasting condition. A proportion of sheep infected with MAP experience active infection involving subclinical or clinical-production effects and mortality detrimental to animal wellbeing and farm economics.

Historically, sheep were commonly used as a convenient ruminant subject for experimental infection studies with MAP, and as a model for bovine PTB [1]. In the last two decades, the use of experimental infection models also became prevalent to better understand infection and disease in countries where Ovine Johne’s Disease was perceived as a problem [2]. Reviewing aspects of experimental and natural infection of sheep with MAP has not been addressed before.

The purpose of this work was to develop a synthesis of the patho-physiology of ovine PTB that could subsequently inform simulation modelling of ovine PTB in a pastoral environment. A PTB modelling approach enables the evaluation of infection dynamics, epidemiology, economics, and possible control strategies within a flock. Such a model should ideally capture mechanisms of infection and transmission including relevant disease stages, shedding levels, force of infection and potential production effects. To our knowledge, only one simulation model of ovine PTB exists in the peer-reviewed literature [3]. Biological assumptions in that spreadsheet model were simplified, to comply with tractability and the general lack of understanding of detailed ovine PTB dynamics at the time; this model thus could not be “taken as an accurate representation of the mechanics of the disease” [3]. We therefore intended to develop an updated synthesis of the current understanding of the patho-physiology of ovine PTB in a way that could directly be used for future attempts to model ovine PTB.

## Assumptions for a candidate model structure

This section synthetizes possible patho-physiological pathways following infection, and identifies infection stages.

### Pathology outcomes following oral exposure to MAP

Understanding the various pathological outcomes of infection with MAP allows identification of stages in disease progression, as compartments of a state-transition model.

#### Infection

A sheep becomes infected with MAP upon ingestion of a dose high enough to cause uptake of the bacterium in the intestinal wall. This is followed by replication, and at least short-term colonisation of the intestine.

In vivo studies, using lamb intestinal loops [4] or ovine macrophage cultures [5] showed that intestinal colonisation occurred within hours of MAP contact. Uptake of 52–86% of inoculum occurred in ovine macrophage cultures within 2 h, for ten different isolates of both C and S strains [5].

This early infection stage is ordinarily referred to as latent infection. It is not necessarily associated with, or predictive of outcomes such as faecal shedding, intestinal histopathology or gross lesions, and clinical signs, making it difficult to recognise. Detecting mere MAP colonization is further hampered by poor sensitivity of tissue culture at this stage of infection, especially towards S strains. After low inoculum doses, tissue PCR positive sheep often have no tissue lesions and are tissue or faecal culture negative [6, 7]. Moreover, early colonization can be transitory in some animals because complete clearance of infection has been observed [8]. Though not commonly reported, tissue PCR appears more sensitive than tissue culture for this stage of infection [9, 10]. Using this sensitive technique, a few studies established that nearly all experimentally inoculated sheep had PCR-positive tissue shortly after challenge [11, 12]. Even low inoculum doses of between 10^3^ and 10^4^ CFU are sufficient to establish intestinal infection, as assessed by tissue PCR, in most artificially challenged sheep [7, 9].

Latent infection does not contribute to infection dynamics because of absence of faecal shedding or production loss. For animals that go on to develop histological lesions, the latent stage can be relatively short with intestinal lesions starting to appear within weeks post-exposure [13]. Hence, this latent stage has little relevance to modelling of PTB in sheep.

#### Pathology

Intestinal lesions of naturally infected sheep are primarily located in the Peyer’s patches of the jejunum and ileo-caecal valve and in the mesenteric lymph nodes. The small intestine, in particular the ileal wall, is more severely affected than efferent lymph nodes [10, 14, 15], and MAP is more abundant in intestinal mucosa than in mesenteric lymph nodes [15, 16]. Hence active infection, in particular in early stages, may be better ascertained by histopathology of ileum and jejunum rather than mesenteric lymph nodes [17, 18].

The severity of lesions determines whether the ileal wall ultrastructure, and therefore its function, is affected. A histological scoring system for ileal lesions was developed by Pérez et al. [19]. Types 1 and 2 are mild lesions consisting of small focal granulomata of epithelioid cells limited to the Peyer patches, while type 2 lesions also extend to the mucosa adjacent to Peyer patches. Type 3 (severe lesions associated with clinical or subclinical disease) present a multifocal to diffuse cellular infiltration, which extends to areas of the mucosa not associated with lymphoid tissues [19]. They extend beyond the lamina propria into the submucosa [16], resulting in a thickening of the intestinal mucosa and atrophy of villi. This alters the capacity of absorption of the small intestine, which is likely to cause clinical disease. Types 3b and 3c lesions, involving diffuse granulomatous enteritis, are the most advanced types [19, 20], and are associated with typical macroscopic lesions at post-mortem. The multifocal rather than diffuse nature of type 3a lesions is evocative of early development of type 3b lesions [19]. Although not as severe as diffuse lesions, and not usually described in clinical cases, they cause an alteration of the ultrastructure of the ileal wall with enlargement of villi and are therefore likely predictive of the development of clinical disease.

The degree of tissue colonization also allows for the differentiation between paucibacillary, presenting no or few acid-fast bacilli (AFB), and multibacillary lesions, presenting abundant AFB [16, 21]. All lesion types are usually scant in AFB, thus paucibacillary, except lesions of type 3b, which are multibacillary. Diffuse pauci versus multibacillary lesions also differ by the nature of the cellular infiltrate. Multibacillary lesions 3b are characterized by a diffuse granulomatous enteritis with massive infiltration of mostly macrophages and epithelioid cells [10, 19, 22, 23], usually with limited lymphocytic and neutrophilic infiltration [16]. These lesions were described historically as lepromatous. On the other hand, the dominant cell type in diffuse paucibacillary lesions 3c is lymphocytic [10, 19], with T cells [22] located mainly within the lamina propria [10, 14]. Paucibacillary 3c lesions were historically referred to as tuberculoid-type [16]. In type 3a lesions, either tuberculoid and lepromatous patterns may be present [21], suggesting a “crucial transition stage” between the two pathogenesis types. Type 3a lesions are thus sometimes also called “borderline lepromatous” [19].

Progression to multibacillary lesions in the terminal ileum appears as an end-stage, irreversible condition [15] associated with permanent faecal shedding of high concentrations of MAP [21, 24–26]. Diffuse intestinal lesions, both types 3b and 3c, are nearly always associated with clinical disease in both naturally and artificially infected sheep [10, 15, 16, 21, 24, 25]. Dissemination to extra-intestinal tissues is associated with multibacillary intestinal lesions [16, 22] and to a lesser extent with paucibacillary 3c intestinal lesions [10]. Noteworthy, in sheep both pauci- and multibacillary types can be pathogenic although it is not clear whether they “represent sequential or divergent stages of PTB” [20]. The simulation model can account for that by incorporating potential production effects and extra-mortality due to PTB for sheep in this advanced disease stage. Sheep can present clinical disease irrespective of MAP abundance in the tissues, but MAP shedding level between pauci- and multibacillary types differs by several orders of magnitude [26]. Hence, the relative contribution of sheep affected with either type to the infection dynamics in the flock is different and this should be accounted for in the model.

#### Faecal shedding

This section pertains to faecal excretion of MAP organisms subsequent to infection and replication in the intestinal tissues, i.e. active shedding as opposed to pass-through. Semi-quantitative faecal shedding data can be obtained using quantitative PCR. With this technique, Kawaji et al. [26] showed that sheep harbouring multibacillary lesions shed about 10^4^ times more femtograms of DNA than the sheep with paucibacillary lesions. In addition to a magnitude difference in the number of organisms shed between sheep presenting pauci- versus multibacillary lesions, in the former, shedding is often only intermittent [18, 27] while that in multibacillary sheep is usually continuous on a daily basis [24]. Excretion was quantified by Whittington et al. [24] using liquid culture and end-point titration, whereupon the total faecal output in four sheep with multibacillary and one with paucibacillary lesions was 6.27 × 10^12^ MAP over 15 days. Considering the contribution of the sheep with paucibacillary lesions as negligible, excretion of MAP for multibacillary animals can be estimated at approximately 1 × 10^11^ MAP per day. Considering that paucibacillary cases shed 10^4^ times fewer and on average only 24% of the time, on a daily basis, the average excretion rate for paucibacillary animals could be estimated at 2.4 × 10^6^ CFU/day.

These shedding figures can inform a mathematical model about the rate of environmental contamination of MAP for compartments of paucibacillary and multibacillary sheep.

#### Synthesis of pathology outcomes

Based on the above observations, the following synthesis is proposed for pathology:

- Latent infection per se does not result in sub-clinical or clinical disease, or production effects. Moreover, evidence to date suggests that latent infection does not play a significant role, if any, in MAP transmission. Hence, there is no justification for its inclusion in a mathematical model of infection dynamics and/or economic effects.
- Animals presenting lesion types 3a, 3b, 3c are a subset of actively infected sheep suffering production losses due to PTB. Potential sub-clinical and clinical effects of PTB and extra-mortality due to clinical PTB should therefore be incorporated in this compartment in a simulation model.
- Paucibacillary lesions are associated with low and possibly intermittent shedding. Multibacillary lesions are associated with high and persistent shedding. Daily shedding rates in corresponding model compartments can be approximated as 10^11^ and 2.4 × 10^6^ MAP/day on average, respectively, for sheep with multi- versus paucibacillary lesions.

### Progression pathways

A number of studies support the existence of two pathological pathways following infection, one leading to recovery and the other to progressive disease. These pathways can be evaluated by multiple testing strategies identifying infection load within tissues, histo- and gross pathology, or shedding into the environment. Confirmation of recovery consists in a lack of progression to clinical disease, regression of lesions and recovery from disease, and clearing of MAP from the tissues.

#### Immunological response

The host immune response is predominantly Th1 cell-mediated in paucibacillary animals, and Th2 humoral in multibacillary animals [19, 28]. This polarisation of immune response exists for a given animal at a given point in time and is controlled by activation of cellular receptors and cytokines [5]. This determines different patterns of T cell activation in the two pathological types [22, 23]. Cytokines such as gamma interferon (IFNγ) and interleukin-10 (IL-10) are thought to be pivotal in establishment or failure, respectively, of a Th1 mediated protective immune response.

Pro-inflammatory IFNγ mediates the adaptive immune response against intra-cellular pathogens [29]. Hence IFNγ response in general is a marker of recent exposure to MAP [30, 35]. High levels of IFNγ are observed in early stages of infection, including latent stages, whereas a decline of IFNγ is predictive of progression to severe pathology, faecal shedding and disease [30]. Genetic expression of IFNγ is up-regulated in intestinal tissues of sheep presenting paucibacillary disease compared with multibacillary or latently infected sheep [22].

On the other hand, IL-10 is an immuno-suppressive cytokine induced by MAP to evade the cell-mediated adaptive immune system, thus allowing the persistence of MAP in macrophages [29, 30]. In early infection, a correlation was noted between an increased anti-apoptotic, anti-destructive response by macrophages infected in vitro, and the survival of MAP in these macrophages [5]. This was mediated by differential cytokine regulation, in particular upregulation of anti-inflammatory IL-10 and down-regulation of pro-inflammatory IL-2. The level of IL-10 increases progressively in infected animals as the disease progresses [30, 31]. However, the precise role of IL-10 is not yet fully elucidated. The cytokine is thought to be associated with the failure of Th1 mediated adaptive immune response and hence is used as a marker of disease progression. However, an elevation of IL-10 levels in peripheral blood can be observed as early as 4 months post-inoculation in experimentally infected sheep [31] and is associated with resistance to disease later in the course of progression [30]. This suggests that the immunosuppressive effect of IL-10 might have a protective effect at the animal level by limiting the damage of the intestinal tissues [29, 30], at least in animals that control the infection. It is not clear however, whether measures of the adaptive immune response adequately reflect the local intestinal immunity [29]. High peripheral blood levels of IL-10 could also result from a failure to act locally. A variation of the adaptive immune response and particularly the expression of IL-10 can be observed in blood versus mesenteric lymph nodes versus ileal tissue. The expression of IL-10 thus clearly differs between sheep with paucibacillary versus multibacillary disease. IL-10 is upregulated in the ileal wall of sheep with multibacillary disease presenting a Th2 dominant response [22]. On the other hand, de Silva et al. [31] observed that the secretion of IL-10 in the mesenteric lymph nodes was lower in multibacillary than paucibacillary lesioned sheep.

This immune polarization was thought to be relatively antagonistic [20]. Thereupon the cell-mediated immune response would correspond to a “controlled” infection observed in early stages and paucibacillary disease. A later switch to a non-protective humoral response would then be associated with multibacillary disease, determining the onset of clinical disease. An excessive cell-mediated immune response reportedly led to advanced inflammation of the intestine walls in severe paucibacillary cases [20]. Thus, paucibacillary infection can also be at the end stage of PTB in sheep, unlike cattle [22]. A switch between Th1 and Th2 responses could be triggered by T cell exhaustion, MAP exposure dose, macrophage bursting size and other host-level metabolic triggers [29].

However, recent research suggests a more complex immunologic response to MAP infection than the classical Th1, then Th2 switch hypothesis. This was based on the observation that half the sheep experimentally infected with MAP actually presented a combined antibody and INFγ response at an early stage of infection [32]. Simultaneous cellular and humoral responses were also observed [11], as well as a lack of early INFγ production in sheep with only focal intestinal lesions. These recent advances in immuno-pathology conclude that progression to disease could result from a generalised failure of the immune system where the Th1 response fails first, rather than switches to a Th2 response.

#### Presence of MAP in intestinal tissues

Detection of MAP organisms in intestinal tissues can be direct, using culture, PCR or a staining method (mostly Ziehl–Neelsen or immunochemistry), or indirect by detecting the presence of microscopic lesions.

Neonatal lambs can develop histological lesions in Peyer’s patches as early as 18 days after inoculation, although such early lesions may not harbour visible MAP [13]. Gilmour et al. [33] determined that MAP was likely actively dividing in intestinal tissue 1 month after inoculation.

In a study using sequential necropsies to describe the chronology of infection/pathology in lambs to 16 months post-inoculation, Kluge et al. [14] first identified AFB in macrophages in lymph follicles of the intestinal wall 1 month post-challenge, along with the first histological lesions. The peak of intestinal lesions corresponded with a sudden peak in the number of AFB detected in tissues, between 4 and 8 months after inoculation. This corresponded with the onset of clinical signs, at 5 and 6 months post-inoculation. The fast onset of extensive multibacillary lesions and clinical signs could be related to the high inoculum dose (10^10^ to 10^11^ MAP) of likely potent MAP from a tissue homogenate. After 16 months, surviving lambs were recovering from disease, presenting healing histological lesions with few AFB.

Similar progression patterns to those above were observed in other experimental studies using sequential necropsies, for inoculum doses around 10^8^–10^9^ of laboratory passaged (attenuated) MAP [8, 33, 34]. Initial infection/pathology established in nearly all sheep necropsied in the first months post-inoculation, followed by a decrease of the intestinal infection load in the first year, and a tendency towards resolution of intestinal pathology in the second year in a proportion of animals. Begg et al. [34] reported that six of 30 sheep surviving 22 months after inoculation had cleared infection, or controlled it below the detection limit. Ten of 30 sheep became clinically affected between 11 and 21 months post-challenge.

Gilmour et al. [8] further examined a subset of five sheep using serial biopsies. Recovery from infection (tissue culture positive turning negative) was directly observed within 27 months in three animals, two of which were clear of histological lesions, while two other sheep progressed to more severe disease. A similar observation was reported by Dennis et al. [15] who serial biopsied tissue from 77 naturally infected sheep. Among the 46 infected, detected by tissue culture, 6 (13%) cleared MAP from tissues between 10 and 36 months of age, while 12 died of clinical disease. Those dying had progressed in less than 2 years from having no intestinal lesions to advanced intestinal pathology. While serial biopsy ostensibly allows disease progression to be monitored, this technique might alter the natural course of the disease.

#### Presence of MAP in faeces

Faecal shedding is more difficult to detect than histo-pathology due to sub-optimum sensitivity of culture, and intermittence of shedding, possibly confounded by shedding levels below the limit of detection. Nevertheless, early onset of transient MAP shedding in faeces can be detected by culture in the first few months post-inoculation in most sheep [27], or even all when using a sensitive PCR [26]. Transient shedding can start as early as 2 months post-inoculation [27]. That study, over 3 to 4.5 years, identified a subgroup of 10 sheep shedding intermittently in the first year, which stopped shedding permanently after a maximum of 16 months post-challenge. These animals might either eliminate MAP infection or control it below the level of detection by faecal culture. Another two sheep were persistent shedders and were the only animals that developed clinical disease, occurring at 20 and 32 months. Using qPCR it is possible to distinguish low and high shedders, which shed quantities several orders of magnitude apart [26]. Sheep shedding high numbers at 13 months post-challenge harboured advanced pathology at post-mortem.

#### Synthesis of progression pathways

Based on the above observations, the following synthesis is proposed for progression pathways:

- Histology, tissue infection and faecal shedding outcomes of MAP infection display a similar pattern of early transient active infection followed by either control or remission, or irreversible progression to clinical disease and high shedding. This dichotomous expression of infection is mediated by polarization of the immune response.
- This can be modelled by sheep entering the active infection stage dividing between a non-progressor pathway, leading to recovery, and a progressor pathway, leading to disease.

### Pass-through of MAP

Pass-through, or “passive shedding”, is usually defined as faecal shedding of MAP without replication in the intestinal wall [35] i.e. in the absence of infection per se. Pass-through of MAP naturally occurs in a heavily contaminated farm environment and has been shown to decrease as heavy shedders are removed from the farm [36, 37]. Experimental data suggests that passing through of orally ingested MAP may occur up to 10 days post-ingestion in sheep [35]. Guidelines to interpret experimental infection models indicate that faecal shedding after 14 days post-inoculation can be interpreted as likely MAP multiplication in the host [38].

Nevertheless, it is difficult to differentiate pass-through per se from early transient (active) shedding, which may also cease later on [27]. Moreover, pass-through of ingested MAP does not preclude true infection to occur at the same time [39]. Repeated exposure to MAP, in particular from the pasture, can result in pass-through of MAP but also later onset of active infection, in cattle and sheep alike [36, 40].

In view of this, Smith et al. [41] considered pass-through as equivalent to latent infection, but this distinction is difficult to prove. This definition corroborates evidence presented in previous sections about ovine paratuberculosis: that even low doses are sufficient to establish infection in most sheep and that latent infection or early shedding can be transient in sheep, followed by recovery from infection. As noted by Mitchell et al. [42], short term transient shedding followed by recovery versus pass-through in the absence of infection have the same result.

In a pastoral environment, pass-through of MAP by exposed animals does not make a difference in the overall level of pasture contamination. However, pass-through could still be a source of infection when considering transmission between paddocks or between farms, if a shedding sheep is introduced into a new environment free of MAP.

### Conclusions and assumptions about pathophysiology for a candidate model

**Figure 1:**
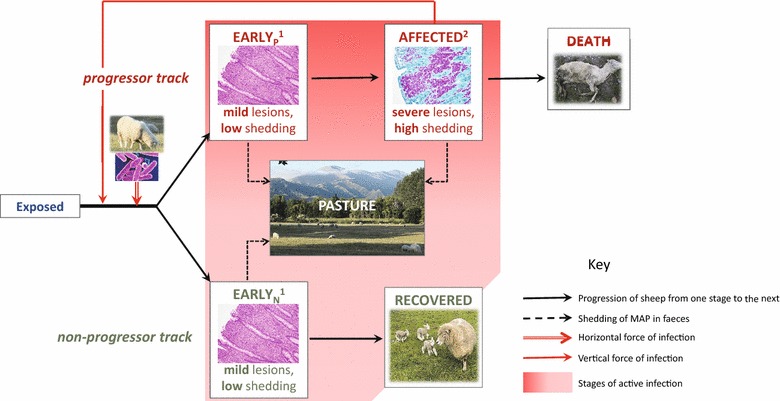
**Structure for a candidate state-transition mathematical model of ovine PTB, based on evidence gathered in this review.** The model represents MAP infection and disease dynamics in a sheep flock in a pastoral environment. The boxes Exposed, EarlyN (early disease in the non-progressor track), EarlyP (early disease in the progressor track), Affected, Recovered and Death each represent a stage of infection/disease. Sheep exposed to MAP progress through successive stages according to the plain black arrows. The box “Pasture” represents the environment in which infectious sheep are shedding MAP (according to the dotted black arrows). The transmission of MAP is represented by the red arrows. The boxes annotated with 1 represent sheep sub-clinically infected with MAP, with no visible production effects. The box annotated with 2 represent sheep affected by MAP, sub-clinically then clinically experiencing production effects. References for the pictures: MAP bacterium [60]; histological lesions in the small intestine [22], Figures 2 and 3; Ewe and triplet lambs: [61]; dead ewe: picture credit Stefan Smith; NZ sheep pasture: picture credit primary author.

The disease stages and pathways identified above were incorporated into a model structure for PTB in sheep, shown in Figure 1 as the basis for simulation of ovine PTB encompassing realistic pathogenic processes. However, due to disparate evidence, low sample size and heterogeneous study design, robust quantitative parameter estimation for a mathematical model was precluded.

Active infection can be measured in experimental studies by histo-pathological changes in the small intestine, and are a proxy for faecal shedding. The presence of severe lesions and/or clinical signs can serve to quantify the proportion of animals entering the progressor pathway towards disease. Shedding data from studies of ovine PTB are less reliable due to intermittence in early stages of progression and imperfect sensitivity, particularly of culture-based methods. Recent techniques of quantitative PCR to detect faecal shedding [43] might partially address this issue in the future. Pass-through of MAP, as such, may not be distinguished from early transient shedding. Latent infection, in the absence of shedding, has no effect on the dynamics of ovine PTB at the flock level and is not represented in the model structure represented in Figure 1.

## Transmission of MAP

This section reviews transmission routes of MAP in natural PTB, to inform the expression of the force of infection in a mathematical model.

The faecal-oral route is the main mode of MAP transmission, which presumably occurs very early in life by MAP exposure from an infectious dam via suckling and from a contaminated environment [28, 35].

### Indirect transmission

In a pastoral environment, indirect transmission via pasture contaminated by infectious sheep drives infection dynamics. Pasture-based transmission is efficient at infecting individual sheep of all ages including adults [40, 44] and spreading MAP on farms previously free of the organism [45, 46].

The quantity of MAP present on pasture is the product of faecal shedding and survival of MAP in the environment. The probability of infection can therefore be modelled via an indirect force of infection driven by the dose of MAP to which susceptible animals are orally exposed by grazing [47].

MAP survival in the environment is a key determinant of this indirect force of infection. In a contaminated pasture, soil, grass and run-off waters are all potentially infectious [48]. MAP survival was observed up to 55 weeks in soil and 24 weeks on grass in fully shaded locations [48] and typically less than 4 months otherwise [49]. Considerable variation of MAP survival between sites and time of the year cannot be fully explained. Nevertheless, shade and vegetation cover considerably increases MAP survival on the soil, while extreme temperature maxima increase MAP decay.

### Transmission to neonatal lambs

In neonates, direct MAP transmission from the dam can occur as vertical and/or pseudo-vertical transmission. A study about vertical transmission specified that the transmission in utero occurred from 1/54 sub-clinically infected ewes and all of five clinically affected ewes [50]. Vertical transmission per se therefore appears a rare event in sheep, virtually limited to clinically affected dams.

On the other hand, pseudo-vertical transmission arises from close dam-to-lamb contact and suckling contaminated udder or milk. MAP organisms were detected in mammary tissues [10] and mammary secretions [50], but only in advanced cases. MAP was also isolated from bulk-tank ewe milk, indicating either excretion in the milk or fecal contamination of the milk [51].

Experimental data supports the dominant role of direct dam-to-lamb transmission prior weaning. In a field experiment, “tracer” ewes from a MAP-free farm were co-grazed with home-bred ewes in a heavily infected flock with high prevalence of Agar gel immunodiffusion test positive animals, just before the lambing season [44]. All lambs were raised from birth in the same contaminated paddock. Onset of infection in lambs was monitored by serial culling and tissue culture. Positive culture appeared earlier in the lambs from home-bred ewes than those from tracer ewes. The cumulative proportion of lambs culture positive up to 8 months of age was significantly higher in the home-bred group, suggesting that the home-bred lambs were exposed earlier and/or to greater challenge than the tracer lambs. Since vertical transmission is seldom observed from clinically normal ewes [50], the apparently higher challenge in neonates from infected dams is likely due to pseudo-vertical transmission. Nevertheless, that experiment also confirms that lambs from tracer ewes can also acquire infection from contaminated pasture prior to weaning [44], highlighting the role of environmental exposure in neonatal infection. That lambs in a pastoral environment can eat substantial amounts of grass from a very early age supports the plausibility of this proposition.

### Synthesis of MAP transmission routes

Based on the above observations, the following synthesis is proposed for transmission:

- In a pastoral environment, infection with MAP mostly occurs via indirect transmission from a contaminated environment, for all sheep irrespective of the age. An indirect force of infection for sheep in all age groups, function of the estimated ingested dose, would account for this infection route in a simulation model. Further quantitative studies are necessary to estimate the transmission parameter of the force of infection.
- Direct dam-to-lamb transmission also occurs in neonates, both from vertical and pseudo-vertical transmission. The frequency of vertical transmission was 100% in clinically affected ewes and 1/54 in sub-clinically infected ewes. This can be included in a mathematical model if the infection status of lambs at birth depends on the dam’s stage of infection. Evidence also suggests that the pseudo-vertical route plays a role in MAP infection dynamics in neonates. This observation, however, comes from low numbers of sheep from an experiment that was not replicated; hence it is difficult to measure and to include in a simulation model.

## Host- and pathogen-level risk factors for infection and disease caused by MAP

The outcome of challenge with MAP may depend upon a number of biological covariates such as the challenge dose, strain, age of sheep at inoculation, breed, number of inoculum doses used, whether organisms were obtained from subculture or directly from tissue homogenates [2, 38], and individual host susceptibility.

### Age at exposure and infection per se

Historically, it was accepted that susceptibility to infection in sheep decreased with age, based on evidence from cattle [35, 52]. However, recent work suggests that older animals can become infected when subjected to high doses and continued exposure [36, 40]. A number of experiments failed to demonstrate a difference in infection rates between sheep infected when young or as adults [6, 15, 40, 44]. Adult sheep between 2 and 10 years old were successfully infected by MAP as assessed by tissue PCR, even with low doses around 10^3^ MAP [6, 9]. Sheep in the high dose group also developed histo-pathological lesions, irrespective of the age at inoculation. Hence, data do not support the assumption of age-related resistance to infection per se.

### Age at exposure and disease

While lambs and adult sheep may be susceptible to infection per se, the proportion developing signs of disease may be related to age.

The number of granulomata in the intestinal tissue of sheep challenged as 1-month-old lambs was significantly greater than in sheep challenged as adults [9]. Lesions extending beyond the intestinal lymphoid tissues into the lamina propria were observed only in lambs [6]. In a high dose group, half of infected lambs (*n* = 8) presented severe, multifocal lesions at post-mortem, while all of 12 infected ewes presented only mild focal lesions.

Similar results were observed following natural infection. McGregor et al. [40] observed that lambs and hoggets developed significantly more and more severe lesions in the intestine, following exposure to contaminated pasture, than sheep exposed as adults. The onset of shedding also occurred earlier in younger sheep. In a longitudinal study of MAP spread in one recently infected flock, only sheep exposed in their first year of age went on to develop detectable shedding, in their 5th year or later [45]. During at least 7 years, “patent infection”, i.e. associated with shedding, remained clustered in those cohorts rather than spread to all age groups in this flock.

Animals exposed to MAP before 2 years of age thus exhibit greater pathological and clinical manifestations due to MAP infection, compared with sheep exposed as adults.

### MAP dose and disease

In an experimental infection model using 3-month-old Merino sheep, a challenge dose of 10 to 10^3^ MAP resulted in negative tissue cultures after 7 and 14 weeks [7] while challenge with 10^8^ MAP resulted in positive tissue culture in all sheep. This indicated that colonization was unlikely to occur at a low inoculum doses. This is in contrast with other studies showing that inoculum doses around 10^3^ did result in at least latent infection in most inoculated sheep [1, 9].

A study about the effect of challenge dose [1] showed that 9 months after challenge, recovery from infection, determined by negative tissue culture, was more frequent in animals challenged with doses around 10^3^ than around 10^9^. Inoculum dose also influences the pattern of subsequent lesions. Doses from 10^3^ to 10^6^ caused focal lesions with a tendency towards resolution [17], or did not cause any lesions [6]. By contrast, inoculation with 10^8^–10^9^ MAP resulted in extensive and severe lesions in most animals [17]. Clinical disease occurred in sheep in the highest challenge group only.

After natural infection via contaminated pastures, McGregor et al. [40] showed sheep on high contamination dose pasture had 3.5 times higher odds of shedding MAP, 18.2 times higher odds of dying of clinical PTB, shed 3.4 times earlier and succumbed to clinical disease 18.6 times earlier than sheep in low exposure paddocks.

These results, from natural and artificial challenge studies, indicate that the outcome of infection differs depending on MAP dose, with low–medium doses resulting in a high proportion recovering from infection/disease, while high doses trigger progression towards severe pathology and clinical disease in most animals.

### MAP strain type and disease

Using RFLP with MAP specific IS900 sequence as a probe, Collins et al. [53] identified 29 strains in New Zealand isolates. These mostly fell into two main strain types, namely sheep (S) and cattle (C), as they appeared to be host species-specific at that time. Further molecular work [54] identified genetic differences between pigmented isolates, corresponding to the sheep strain, and non-pigmented isolates, corresponding to the cattle strain. Those authors introduced a new nomenclature to classify strains based on phenotypic and genotypic characteristics. Strain type I comprises a group of organisms difficult to grow in culture and showing a strong host preference for sheep, which may be more virulent for sheep whereas strain type II comprises organisms easy to grow in culture, commonly isolated from cattle, but also from a broad range of species including deer and sheep [54]. An additional type (III) was later identified by whole genome sequencing as a subtype of S strain [55]. Type III MAP are found in natural cases of ovine PTB in Spain for example, and have been successfully used in experimental challenge studies [11, 12].

The relative difficulty to grow S strains [56] and the lack of mixing opportunities between cattle and sheep strains [57] might contribute to the apparent species-specificity of the S strain, while the C strain shows no host preference [55]. However, S strain isolates were identified from cattle in Australia and Iceland [56]. Moreover, a recent study in New Zealand demonstrated that 80% of infected beef herds and 10% of infected dairy herds were infected by S strain [58]. The authors concluded that transmission of MAP between livestock species is frequent in a pastoral context where co-grazing is common.

In the absence of strict species specificity between strains, it is important to understand potential differences in the host–pathogen interplay and subsequent disease [55]. Histo-pathological examination revealed extensive and diffuse intestinal lesions with abundant AFB after 5 months in sheep inoculated with a S strain from a gut homogenate [59]. By contrast, sheep inoculated with passaged C strains developed focal lesions after 5 months, mostly located in the mesenteric lymph nodes. One group inoculated with a C strain from a tissue homogenate, thus directly comparable with the S strain, developed mostly intestinal lesions, “showing a diffuse character in some parts”, thus the severity resembled that of the tissue homogenate S strain inoculum group. Nevertheless, the authors’ conclusion was that the C strains induced more focal lesions while the S strains induced lesions of multi-focal or diffuse character. Looking over a longer period of time, Fernandez et al. [12] reported that after 5 months, sheep infected with passaged C strains showed more severe and diffuse disease in the small intestine than those infected with passaged S strains. However, the lesions induced by the C strains tended to regress in extent and severity after 1 year, unlike those induced by the S strains, thus resembling the pattern observed in [59]. In both these studies, the presence of some extent of fibrous granulomata induced only by C strains was suggestive of the regressive character of these lesions. This fibrous character was also observed in vaccinated sheep [20]. A significantly higher IFNγ response was noted in sheep infected with the C strain, versus a lower [59] or inconsistent [12] IFNγ response with the S strain. Stewart et al. [27] also reported a lower and shorter IFNγ response associated with S strain than with C strain, concluding that S strains may be less pathogenic in sheep.

Altogether, these observations suggest that infection with C strains triggers a stronger cellular immune response associated with potential tissue damage observed in some, but not all animals, but also leading to disease resolution. Based on that, Fernandez et al. [12] speculate that most sheep exposed to a C strain might recover from infection. Moreover, a typical feature of lymph node granulomata induced by C strains was the presence of central caseous necrosis with Langhans-type giant cells [12, 59]. This feature was noted elsewhere in experimental ovine PTB [20] but is not typical of naturally occurring infection, presumably attributable to S strains [19]. This could therefore be typical of ovine PTB induced by a C strain.

With respect to clinical disease, Stewart et al. [27] observed the same cumulative clinical disease incidence (1/10 sheep) following inoculation with either strain during 3 years of follow-up. Similar shedding patterns in those groups were also apparent, although statistical power was limited by a small sample size.

To summarize, experimental evidence to date, albeit limited, suggests a different lesion pattern of ovine PTB with S strains than C strains. However, the significance of this finding for subsequent pathology severity and progression to disease is unclear [55] and more research is needed.

### Inoculum type and progression

MAP organisms obtained both after culture and direct from tissue homogenate have been used in experimental challenge studies. Virulence attenuation by laboratory passage has been observed frequently in experimental studies.

Begg et al. [34] showed that challenge with tissue homogenate caused “higher levels of infection and disease” than challenge with a pure, low passage culture, despite a 20 times higher dose in the pure culture. After continuous passage (over 10), neither infection nor histopathology were observed despite that sheep were dosed with 10^9^ organisms. Verna et al. [59] also observed milder histo-pathology with passaged organisms while intestine homogenate inoculum induced more severe and more extensive lesions. Sheep inoculated with passaged MAP shed only transiently 2 months post-inoculation, then stopped, while sheep inoculated with intestine homogenate shed transiently for several months or even became permanent shedders in one experiment [27]. A proportion of sheep in the latter group developed clinical disease. Since MAP dose was not determined for the tissue homogenate inoculum, dose is a possible confounder for those observations. In an experiment where homogenate and passaged inocula were enumerated, with animals given either approximately similar doses around 10^8^ MAP, no significant difference between groups were observed in the proportion progressing to disease after 13 months [26]. However, the sheep inoculated with homogenate started shedding and had clinical signs earlier than those given passaged culture inoculum [18].

A more recent study, involving 14 sheep, was designed to assess virulence attenuation due to in vitro passage [11] and showed significantly higher IFNγ response in the group dosed with tissue homogenate. In the group challenged with passaged MAP, the IFNγ response was no different to that of the controls. The homogenate inoculum induced more severe and extensive lesions than passaged MAP, at a comparable dose.

Hence, passage of MAP in vitro, induces a significant attenuation of virulence. However, one drawback of inoculating direct tissue homogenate is the difficulty to enumerate the inoculum dose. These elements need to be considered in designing and interpreting the results of experimental infection models for PTB.

### Synthesis on the effect of host- and pathogen-level risk factors

Based on the above observations, the following synthesis is proposed for the effect of host- and pathogen level risk factors:

- Age at exposure, dose, strain and inoculum type all influence infection and/or progression to disease.
- These effects confound each other at the study-level, making it difficult to tease them apart in individual studies.
- Confounding due to host and pathogen-level features therefore limits the potential to estimate parameters from individual studies to inform simulation modelling.

## Conclusion

The review addresses current knowledge about pathophysiology of ovine PTB. This knowledge was synthetized to inform a candidate model structure for ovine PTB (Figure 1). The outcomes observed after experimental infection were usually comparable with those observed in natural paratuberculosis, however attenuation of MAP virulence upon laboratory passage represents an artefact in some experimental trials.

Pathogenesis of ovine PTB is driven by the development of active infection involving pathology in the small intestine, while latent infection has little clinical or epidemiological relevance. Progression to clinical disease is associated with the severity and extent of intestinal lesions. Paucibacillary and multibacillary lesion types can both lead to clinical disease. The presence of histological lesions in the intestine appears a good proxy for shedding of MAP and vice versa. Recovery from infection and from sub-clinical disease is frequent.

Transmission in a pastoral environment is driven by MAP dose in the pasture, including for lambs pre-weaning. Pseudo-vertical transmission also plays an important role. Vertical transmission per se appears to be rare in sheep and is limited to clinical cases. These observations can be used to inform the expression of the force of infection in the mathematical model, despite the fact that quantitative transmission parameters cannot be derived from this review.

Older animals appear susceptible to infection but relatively resistant to progression to disease. Higher MAP doses are both more infectious and more pathogenic, as is inoculation with homogenate rather than laboratory-passaged MAP. The relative pathogenicity of C and S strains in sheep remains unclear.

The outcome of individual experimental challenge studies is often confounded by factors such as age, dose or inoculum type. This makes it difficult to compare outcomes between studies. It was thus impossible to estimate quantitatively the magnitude of the effects demonstrated in this review. Further research is needed to contribute to quantitative parameter estimation for the model presented in Figure 1.

## Abbreviations

IFN: interferon
IL: interleukin
MAP: *Mycobacterium avium* subsp. *paratuberculosis*
PCR: polymerase chain reaction
PTB: paratuberculosis
